# Unravelling mechanisms regulating Mincle activation

**DOI:** 10.1038/s42003-025-09493-8

**Published:** 2026-01-08

**Authors:** Lindsay G. Serene, Robert Buchanan, Alex J. McCarthy, Alexiane Decout

**Affiliations:** 1 Centre for Bacterial Resistance Biology, Department of Infectious Diseases, Imperial College London, London, UK; 2 Division of Biomedical Sciences, Warwick Medical School, University of Warwick, Coventry, UK

**Keywords:** Pattern recognition receptors, Inflammation, Adjuvants

## Abstract

Mincle is a C-type lectin and potent driver of inflammation. Given Mincle’s ability to recognise a complex repertoire of endogenous and exogenous ligands, a network of regulatory mechanisms that precisely control Mincle activation is required to prevent excessive inflammatory responses. Here we discuss the mechanisms that govern Mincle-dependent cellular activation and the gaps in our current knowledge. Understanding the key mechanisms regulating Mincle activation could pave the way for the rational design of targeted immunotherapies and vaccines adjuvants.

## Abstract (web summary)

This review discusses what is known about Mincle regulation: where and when it is expressed, how it binds its ligands, and the factors involved in modulating its signaling.

Mincle (Macrophage-inducible C-type lectin; *CLEC4E*) is an immune receptor that plays a crucial role in the innate immune system, as it can recognise and respond to pathogens and damaged host cells or their components^1–3^. Despite Mincle gaining increasing attention for its roles in immune responses, our understanding and appreciation of the mechanisms that regulate Mincle activation are incomplete. This is important knowledge given that Mincle activation is crucial for fine-tuning immune responses and understanding pathogenesis of diseases linked to Mincle dysregulation. Furthermore, the discovery of Mincle as the receptor mediating the adjuvant effect of Freund’s Adjuvant^4,5^ in 2009^6–8^ and its identification as a key target in vaccine adjuvant development has sparked the generation of a broad range of Mincle ligands and formulations as vaccine adjuvants^9–13^. Understanding the key mechanisms regulating Mincle activation could pave the way for the rational design of targeted immunotherapies and vaccines.

Like other C-type lectin receptors, Mincle has an extracellular carbohydrate recognition domain (CRD) that senses endogenous and exogenous ligands (Fig. 1A). However, Mincle stands out among the C-type lectin receptors by the complex repertoire of ligands it can detect. A recent study suggests that this broad specificity was acquired through evolution; Mincle being originally a self-recognising receptor that evolved to detect pathogen-associated molecular patterns (PAMPs)^14^. Mincle possesses an extended carbohydrate binding site composed of a conventional EPN motif at residues 169–172, a secondary carbohydrate binding site and two hydrophobic grooves adjacent to the primary binding site. Three of these four binding sites need to be occupied for effective recognition of the ligand^12,15^. Mincle’s extended carbohydrate binding site can accommodate a range of glycolipids from bacteria and fungi including mycobacteria^7,12,16^, *Streptococcus pyogenes*, *Streptococcus* pneumoniae^17,18^, *Staphylococcus aureus*^19^, *Lactobacillus plantarum*^20^, *Malassezia furfur*^21^ and *Cryptococcus neoformans*^22^. Furthermore, human, but not murine, Mincle has a cholesterol recognition amino acid consensus (CRAC) motif allowing the detection of host and bacterial-derived cholesterol and cholesterol derivatives^23–25^. Additionally, proteins can also be detected by Mincle through an unknown ENP-independent mechanism, as demonstrated by a small protein called SAP130 that is secreted upon necrosis of mammalian cells^26,27^ (for an extended review about recognition of Mincle ligands, see Braganza et al. 2018^28^).

**Fig. 1:**
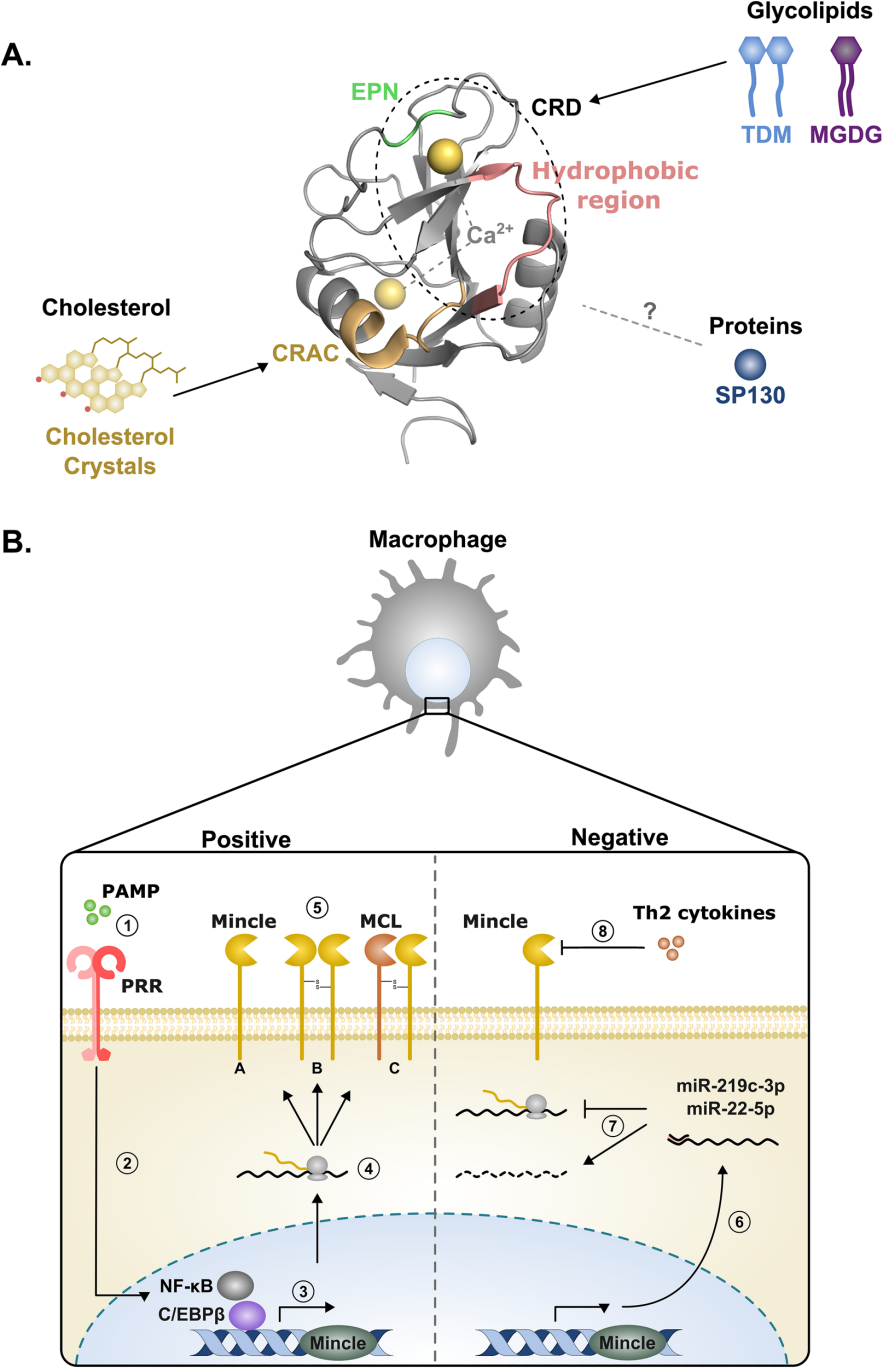
Ligand binding sites and regulation of Mincle expression. **A** Mincle interacts with a diverse range of ligands including, glycolipids, proteins, and cholesterol. These interactions are mediated by different regions of the Mincle ectodomain, modelled here with PyMOL Version 3.1.6.1 (PBD: 3wh2). For example, glycolipids (*M. tuberculosis* TDM and *S. pyogenes* MDGD) interact through associations with a conserved EPN sequence and proximal hydrophobic regions in the CRD, whereas cholesterol crystals bind to the CRAC motif. For other ligands, like Sap130, the binding site remains unknown^24,28,40^. **B** Mincle expression is limited to a small subset of cells, including macrophages. Its expression on the cell surface is inducible and positively regulated by several factors. Sensing of pathogen molecular patterns (PAMPs) by pattern recognition receptors (PRR)(1) induces the translocation of transcription factors NF- κB and C/EBPβ into the cell nucleus (2). This drives the transcription (3) and translation of Mincle mRNA (4). Mincle can then be translocated to the cell surface (5) as a monomer (**A**), homodimer (**B**), or heterodimer with MCL (**C**). Mincle expression is also negatively regulated by microRNAs (6-7) and Th2-type cytokines (8). MicroRNAs, miR-219c-3p and miR-22-5p, bind the 3’ UTR of Mincle mRNA to block translation (6) and lead to its degradation (7). Similarly, Th2-type cytokines have been shown to downregulate Mincle expression in a STAT6-dependent manner (8), restricting its expression to pro-inflammatory states. TDM trehalose-6,6′-dimycolate, MDGD monoglucosyldiacylglycerol, CRD carbohydrate recognition domain, CRAC cholesterol recognition amino acid consensus, NF-κB nuclear factor kappa-light-chain-enhancer of activated B cells, C/EBβ CCAAT/enhancer binding protein β, MCL macrophage C-type lectin, STAT6 signal transducer and activator of transcription 6.

Given that Mincle recognises a diverse range of endogenous and exogenous ligands and is a potent driver of inflammation, a network of regulatory mechanisms that precisely control the binding of Mincle to its ligands and the subsequent signalling cascades is required to prevent excessive inflammatory responses. In this review, we discuss what is known about Mincle regulation: where and when it is expressed, how it binds its ligands, and the factors involved in modulating its signalling (Box 1). We highlight what remains unknown and how these gaps might be addressed to provide a more comprehensive understanding of the multi-faceted ways in which Mincle regulation contributes to health and disease.

## Regulation of Mincle expression

Control of cellular Mincle expression is the first regulatory mechanism (Fig. 1B). Mincle is absent from most cell types and generally expressed on myeloid immune cells in both humans and mice, particularly macrophages^17,18,27,29,30^, dendritic cells (bone marrow-derived)^25,29,31^, and neutrophils^18,29,32^. However, Mincle expression is typically very low on non-stimulated myeloid immune cells. Higher Mincle expression is induced in cells upon immune stimulation, likely occurring to promote Mincle detection of pathogens and activate downstream signalling pathways. Thus, basal Mincle expression is kept at a minimum and increases in the presence of danger signals through the influx of myeloid immune cells and upregulation of Mincle expression.

The inducible nature of Mincle was first demonstrated by Matsumoto et al. in 1999, who showed that Mincle transcript and protein expression were upregulated by several inflammatory stimuli including IFN-γ, IL-6, TNF-α, and LPS^33^. In contrast, the Th2 cytokines IL-4 and IL-13 have been shown to induce downregulation of Mincle expression in monocytes, macrophages, dendritic cells, and neutrophils^12,31,34^. This work highlights that Mincle expression may be restricted in non-inflammatory environments and upregulated in inflammatory environments^35,36^. Tight control of Mincle expression by a subset of immune cells and under specific conditions has fueled the identification of the molecular mechanisms driving its expression, including downregulation of Mincle mRNA via miRNA repression, upregulation via activation of transcription factors and translocation of Mincle protein to the cell surface by the formation of dimers with other immune receptors.

Repression of Mincle expression can be mediated by microRNAs (miRNAs) that bind to messenger RNA (mRNA) resulting in mRNA degradation and inhibition of translation. Indeed, a miRNA called miR-219c-3p interferes with translation of Mincle mRNA transcripts to prevent Mincle expression in macrophages in the absence of danger signals^26^. Similarly, miR-22-5p miRNA represses translation of Mincle mRNA transcripts and cellular Mincle levels^37^. Both miRNAs bind to the 3′ UTR of Mincle transcripts. This highlights an important role for miRNA-mediated repression in regulating Mincle-mediated cellular activation and immune responses. It also indicates that the downregulation of Mincle-targeting miRNAs, under immune stimulation, provides a mechanism to increase Mincle expression and amplify Mincle-dependent immune responses^26^.

Inducible Mincle expression is driven through activation of pattern recognition receptors (PRRs), their signalling pathways, and downstream transcription factors. The best-defined trigger is the TLR4 ligand LPS, which induces a MyD88-dependent upregulation of Mincle expression^38^. Schoenen et al. have shown that LPS-induced Mincle expression promotes activation of the early growth response transcription factor C/EBPβ^39^. Importantly, this regulation of expression was demonstrated to regulate Mincle-dependent functions, as C/EBPβ deficient macrophages and dendritic cells did not upregulate Mincle and were unable to mount a response against a Mincle agonist, the mycobacterial cell wall component trehalose-6,6′-dimycolate (TDM)^39^. Thus, C/EBPβ likely provides a major mechanism to upregulate Mincle-dependent immune sensing and functions following initial priming of cells by the sensing of PAMPs and/or DAMPs.

Finally, Mincle expression can be regulated by an alternative C-type lectin receptor called macrophage C-type lectin (MCL; *CLEC4D*). MCL is structurally very similar to Mincle, detects TDM and signals through FcRγ^40^, but it is constitutively expressed on most myeloid cells^41^. Importantly, MCL was shown to form heterodimers with Mincle and to facilitate Mincle translocation to the cell surface, thereby positively regulating Mincle expression^42,43^.

## Ligand binding and activation of the signalling pathways

Whilst expression of Mincle controls cell surface availability, the functional activation of Mincle is dependent on the specific recognition of its ligands. Like most lectins, Mincle monomers form relatively low-affinity interactions with their ligands. It can be hypothesied that this phenomenon provides a mechanism that prevents Mincle induced activation and inflammation in environments with a low abundance of ligands.

Increasing ligand affinity through receptor oligomerisation is a widely shared feature among C-type lectin receptors. Mincle has been shown to form homodimers, both in solution and in cells^44,45^. These dimers are stabilised by a disulfide bond in the neck region of Mincle and are detected at steady state, in the absence of ligand^45^. Mincle also forms heterodimers with MCL but how these heterodimers are formed is unclear^42,46^. Since Mincle homo- and hetero dimerisation is observed in the absence of ligands, dimerisation is unlikely to directly drive activation of downstream signalling pathways but rather (i) promote Mincle expression, as previously discussed^42,46^, or (ii) enhance the affinity of Mincle for large glycolipids, driving Mincle specificity^42^. Indeed, very long fatty acids such as the 90 carbon-long mycolic acids of TDM cannot be accommodated in the hydrophobic pockets of a Mincle monomer^12^. Since the capacity of MCL to interact with several Mincle ligands is unknown, it is unclear whether the preferential formation of homo- or heterodimers could bias the sensitivity of Mincle towards a specific subset of ligands.

Interestingly, we and others have observed that Mincle binding does not directly correlate with signalling^12,47,48^, suggesting that other parameters such as multivalency and ligand presentation play a key role in Mincle activation. Although never formally demonstrated, higher order oligomerisation of Mincle seems to be required to cluster Mincle dimers on the cell surface and activate cellular signalling. Indeed, Mincle ligands are typically tested for cell activity when immobilised^6,7,9,12^. A water-soluble trehalose dibehenate (TDB) analogue induced strong Mincle signalling in a reporter cell line but moderate cytokine production from granulocyte-macrophage colony-stimulating factor (GM-CSF) treated mouse bone marrow cells. Notably, the analogue failed to promote phagocytosis (an important second signal for inflammasome activation) or an IL-1β response. Dynamic light scattering analysis showed that at concentrations above 1 µM, TDB formed micelles, whereas the analogue did not, providing support for the ligand multivalency hypothesis in Mincle-mediated phagocytosis^47^. Beyond increasing the affinity of the ligand, we and others have hypothesised that multivalency is required for signal transduction^9,24^. The ‘phagocytic synapse’ theory, demonstrated for Dectin-1 signalling^49^, is an attractive mechanism considering the similarity between Mincle and Dectin-1 signalling pathways^9^. Upon interaction with particulate ligands, Dectin-1 clusters at the contact site, leading to the formation of a ‘phagocytic synapse’ which is analogous to the ‘immune synapse’ assembled between T-cells and antigen-presenting cells (APCs). Dectin-1 clustering expels the regulatory phosphatases CD45 and CD148 to the periphery of the synapse, creating a ‘bullseye’ pattern. Exclusion of CD45 and CD148 does not occur in response to the binding of soluble ligands because they do not trigger adequate receptor clustering to isolate the phosphatases^49^. Whether this mechanism underlies the multivalent ligand presentation requirement of Mincle remains to be investigated.

Furthermore, modes of multivalent presentation influence Mincle-mediated immune responses. Stocker et al. compared micellar, plate-coated, and bead-coated trehalose esters and observed a significant effect on the mode of presentation. Plate coated ligands induced stronger IL-6 and IL-1β production from bone marrow-derived macrophages than the micellar formulations, and increasing the diameter of the beads coated with TDB enhanced cytokine production. This may suggest a key role of phagocytosis in promoting Mincle signalling. Interestingly, not all trehalose esters were equally sensitive to the mode of presentation^50^. Finally, higher particle coating density also increased Mincle activation, as demonstrated with aminopropyl silica nanoparticles (A-SNPs) coated with brartemicin-derived trehalose glycolipid (UM-1098)^9,51^.

Overall, the chemical structure and multivalent presentation of Mincle ligands strongly impact their ability to activate downstream signalling pathways. Yet, our understanding of the mechanisms underlying the requirement for multivalent presentation and how ligand density and 3D presentation modulates downstream signalling remains superficial. This gap needs to be filled to accelerate the development of new Mincle agonists.

## Mincle signalling via ITAM and ITAMi

**Fig. 2:**
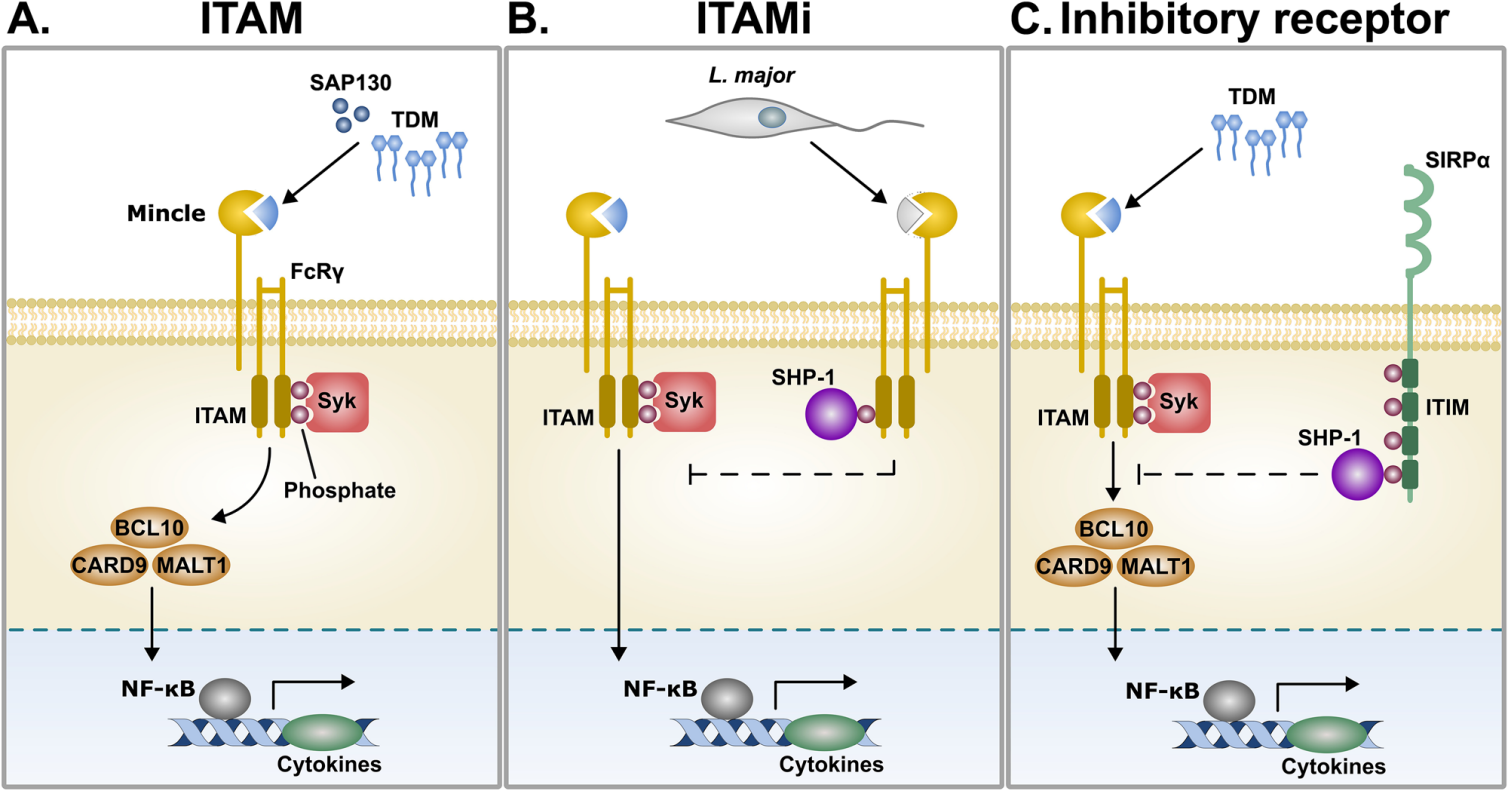
Regulation of Mincle signalling. **A** Mincle associates with FcRγ, which encodes an immunoreceptor tyrosine-based activation motif (ITAM) domain in its cytoplasmic tail, to signal. In classical ITAM mediated signalling, crosslinking of Mincle with its ligand (e.g., mycobacterial TDM or SAP130) leads to the phosphorylation of ITAM tyrosine residues, recruitment tyrosine protein kinase Syk, formation of the CARD9/MALT1/BCL10 signalling complex, and transcription of pro-inflammatory cytokines. **B** Mincle has also been shown to transmit inhibitory ITAM signals (ITAMi), including following binding to an unknown, soluble Leishmania ligand. Mincle-mediated ITAMi signalling dampens pro-inflammatory ITAM signalling and contributes to parasite immune evasion. **C** Mincle signalling can also be negatively regulated by ITIM-bearing inhibitory receptors, such as SIRPα. Crosslinking of inhibitory receptors leads to the recruitment of phosphatases, such as SHP-1, that de-phosphorylate downstream signalling molecules involved in classical ITAM-mediated pro-inflammatory cytokine responses, thereby dampening Mincle-mediated signalling. FcRγ Fc receptor gamma chain, TDM trehalose-6,6′-dimycolate, SAP130 Sin3A associated protein 130, Syk spleen tyrosine kinase, CARD9 caspase recruitment domain-containing protein 9, MALT1 mucosa-associated lymphoid tissue lymphoma translocation protein 1, BCL10 B-cell lymphoma/leukemia 10, SIRPα, signal regulatory protein alpha.

Emerging evidence indicates that detection of Mincle ligands can trigger both activating and inhibitory ITAM-dependent signalling pathways. Classically, following binding of its ligand, Mincle associates with the Fc receptor gamma chain (FcRγ) and potentiates a well-defined signalling cascade that drives the production of proinflammatory cytokines^27^. Briefly, the cytoplasmic tail of FcRγ has two immunoreceptor tyrosine-based activation motifs (ITAMs) that each encode a conserved 4 amino acid sequence that includes a tyrosine residue (Fig. 2A). Crosslinking of Mincle leads to the recruitment of Src family kinases and phosphorylation of the ITAM tyrosine residues^27,52^, which serve as important docking sites for spleen tyrosine kinase (Syk). Syk further phosphorylates downstream signalling molecules and leads to the formation of a CARD9/MALT1/BCL10 complex and translocation of the transcription factor NF-κB into the cell nucleus. This drives the transcription of pro-inflammatory cytokine-encoding genes such as TNFα, IL-6, and IL-23 and contributes to a range of effector functions including ROS production, potassium efflux, NLRP3 inflammasome activation, and Th1 and Th17 polarization^53–55^.

However, ITAM domains have also been revealed to be able to transmit inhibitory signals, subsequently called inhibitory ITAMs (ITAMi; Fig. 2B). Since the ITAMs in FcRγ can transmit inhibitory signals, this raises the prospect that Mincle-induced ITAMi signalling may regulate Mincle-induced cellular activation. This has been observed in dendritic cells following exposure to *Leishmania major*, one of the parasites responsible for Leishmaniasis. During infection, the parasites secreted an unidentified soluble ligand that induced Mincle ITAMi signalling and delayed the detection of Leishmania parasites by dendritic cells. While Mincle expression was dependent on MCL, its signalling was MCL-independent as recombinant MCL did not bind soluble Leishmania extract and was dispensable for Mincle mediated inhibitory signalling in murine bone marrow-derived dendritic cells^56,57^. Much remains unknown about the mechanisms regulating the transition from Mincle ITAM to ITAMi signalling following Leishmania infection. However, the authors hypothesised that MCL-independent binding between Mincle and its Leishmania ligand may be weaker than interactions mediated through the Mincle-MCL heterodimer, and this weaker signal triggered a transition to ITAMi^57,58^. This hypothesis is supported by research showing that low-avidity interactions between other activating receptors, including FcαRI, and their ligand can lead to the incomplete phosphorylation of cytoplasmic ITAM domains^58^. This hypophosphorylation subsequently results in the recruitment of SHP-1 phosphatases, dephosphorylation of nearby activating receptors, and dampening of the immune response^57^. These findings underscore the importance of understanding how, when, and what initiates Mincle-mediated inhibitory ITAM signalling. Structural studies of Mincle receptors bound to low and high avidity ligands could provide insight into the interactions driving ITAM versus ITAMi signalling pathways^59^. Additionally, high-throughput screens for low-avidity interactions using Avidity-based Extracellular Interaction Screening (AVEXIS)^50^ or cell-based screening methods for SHP-1 and FcRγ ITAM domain interactions could provide insight into the mechanisms driving the regulation of Mincle signalling toward activating versus inhibitory pathways.

## Modulation of Mincle-induced signalling

As inhibitory receptors are expressed on the same cell populations as Mincle, it can be speculated that activation of intracellular immunoreceptor tyrosine-based inhibitory motifs (ITIMs) in inhibitory receptors provides a mechanism to regulate Mincle-dependent signalling of FcRγ via its ITAM motifs, cellular activation, and functions (Fig. 2C). Inhibitory receptors are negative regulators of cellular activation that promote immune homeostasis^60^. A hallmark of inhibitory receptors is the presence of ITIMs in their cytoplasmic tails. These motifs are composed of a conserved sequence of amino acids: S/I/V/LxYxxI/V/L, where x is any amino acid, and Y is a tyrosine residue. Crosslinking of inhibitory receptors with their endogenous ligands, including MHC molecules, sialic acid, galectins, glycolipids, and extracellular matrix proteins, induces phosphorylation of ITIM tyrosine residues, and leads to the recruitment of SH2-domain-containing phosphatases that dephosphorylate molecules necessary for the downstream signalling of activating receptors that signal through ITAMs. Indeed, several inhibitory receptors have been shown to modulate FcRγ-mediated effector functions on neutrophils and macrophages^61–65^. This results in the dampening of activating receptor effector functions such as pro-inflammatory cytokine responses, phagocytosis, and ROS production^63^. Together, this information opens the question of whether inhibitory receptors regulate Mincle-dependent signalling events and functions.

Recent research provides some evidence supporting the hypothesis that inhibitory receptors provide a mechanism to negatively regulate Mincle-dependent signalling^66,67^. In a first study, TDM stimulation of murine bone marrow macrophages was shown to induce the formation of a Syk-dependent functional signalling pathway but also induced the activation of CD11b and recruitment of the inhibitory SIRPα receptor. SHP-1 was subsequently shown to dock onto SIRPα and dephosphorylate Syk to negatively-regulate Mincle activation and secretion of proinflammatory cytokines TNF-α and IL-6. Indeed, *Sirpα*−/− bone marrow macrophages secreted significantly higher levels of both cytokines as compared to wildtype bone marrow macrophages following TDM stimulation^66^. A second study found that a non-glycosylated subclass of mycolic acid interacts with the inhibitory CLEC12A receptor. Importantly, *CLEC12A*−/− mice (Mincle + ) have an enhanced host immune response following mycobacterial infection compared to wildtype mice (Mincle+ and CLEC12A + ), including elevated secretion of the chemokine MCP-1. Thus, these assays reveal that interaction of non-glycosylated mycolic acid expressed on the bacterial surface with CLEC12A likely dampens TDM-induced Mincle activation^67^. The results of these studies provide evidence that inhibitory receptors can act to regulate Mincle function and modify Mincle-dependent cellular responses.

Whilst our understanding of Mincle regulation by inhibitory receptors is in its infancy, previous research indicates they play an important role in fine-tuning FcRγ-mediated signals and activation. Given the large number of inhibitory receptors expressed on myeloid cells, identifying when and which inhibitory receptors co-express with Mincle by RNAseq analysis could pave the way for the rationale design of mechanistic studies to identify which inhibitory receptors regulate Mincle activation. In vivo murine models may provide additional insight into the biological relevance of inhibitory receptor signalling on Mincle regulation. However, differences between human and murine inhibitory receptor structure and ligand interactions, as well as the fact that some bacteria known to interact with Mincle are obligate human pathogens (*M. tuberculosis*, *S. pneumoniae*), often requires the generation of transgenic mouse lines to permit mechanistic studies^68^. Together, these and other approaches would help form a more complete understanding of inhibitory receptor-mediated Mincle regulation.

## Conclusions & Perspectives

Mincle is a dynamically expressed receptor and potent inducer of immune responses upon sensing of exogenous and endogenous molecular signals. These include, but are not limited to, cellular differentiation and activation, phagocytosis, and ROS and proinflammatory cytokine production. Because of its role in mediating the effects of Freund’s Adjuvant, Mincle is also a valuable target of future vaccine adjuvants. Understanding the mechanisms that regulate when Mincle can sense and drive immune responses against exogenous and endogenous molecules is critical to understanding health and disease; however, important gaps remain.

The expression of Mincle is maintained at a minimal level in a healthy context, and upregulated following the detection of potential threats^26,39,42^. One remaining gap in knowledge is the regulation of the expression of signalling elements downstream of Mincle. Some cells fail to activate downstream signalling despite expressing high surface levels of Mincle^12^. This may indicate that expression of downstream signalling elements is also tightly regulated in a cell type, and possibly stimulus, dependent manner, which remains to be delineated. Mincle expression levels also vary widely from one person to another^29,69^. Thus, understanding how Mincle surface expression and signalling is regulated is an important consideration when developing and maximising the efficacy of Mincle-targeting small molecules.

Whilst it has been empirically shown that the multivalent presentation of ligands is likely required for Mincle activation by glycolipids, the 3D requirement of multivalent interactions on Mincle binding and subsequent signalling are not fully understood. Furthermore, it remains unknown how some non-glycosylated ligands such as SAP130 bind Mincle and seem to bypass this multivalency requirement. Many groups are currently developing different modes of formulation, including liposomes, emulsions and nanoparticles, and controlled 3D scaffolds that will provide valuable insights toward the optimal presentation of Mincle ligands with preserved inflammatory properties^9,47,50,51,70^. Improved formulations will yield valuable mechanistic insights and will be a major step toward therapeutic translation.

Better understanding of Mincle signalling is also integral in moving toward therapeutic translation. This is especially relevant as emerging evidence reveals Mincle signalling is intricately regulated by multiple mechanisms, including the transition from activating ITAM to inhibitory ITAMi signalling pathways and the dampening of Mincle-mediated activating signals by inhibitory receptors^57,71,72^. These mechanisms of regulation raise important questions of whether other ligands shift Mincle-FcRγ from activation to inhibitory configurations as a form of regulating Mincle-induced immune responses and how these interactions could inform the design of new therapeutics or vaccine strategies^66,67^. They also underscore the need to identify which other human inhibitory receptors regulate Mincle-ITAM-Syk cellular signalling and the mechanisms involved^60,72^.

In conclusion, current literature suggests that Mincle surface expression and signalling are tightly regulated to aid the immune response and limit damage caused by excess inflammation. A more comprehensive mechanistic characterisation of how these events are controlled in health and disease would inform the development of more efficacious Mincle-targeting small molecules and vaccine adjuvants.

### Reporting summary

Further information on research design is available in the Nature Portfolio Reporting Summary linked to this article.

## Supplementary information

Reporting summary
